# Supplementary figures and images for: Experimental lung injury induces cerebral cytokine mRNA production in pigs
Source: PeerJ. 2020 Dec 9;8:e10471. doi: 10.7717/peerj.10471 (PMC7733330; doi:10.7717/peerj.10471)

A

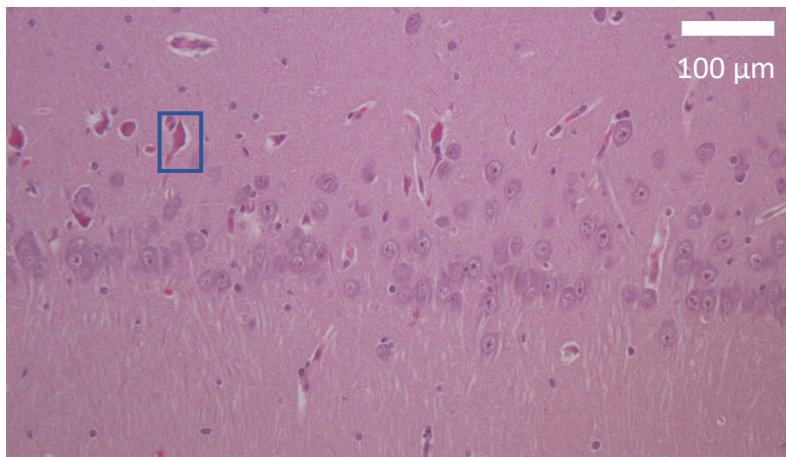

pyknosis and  
eosinophilic  
degeneration  
of a neuron

B

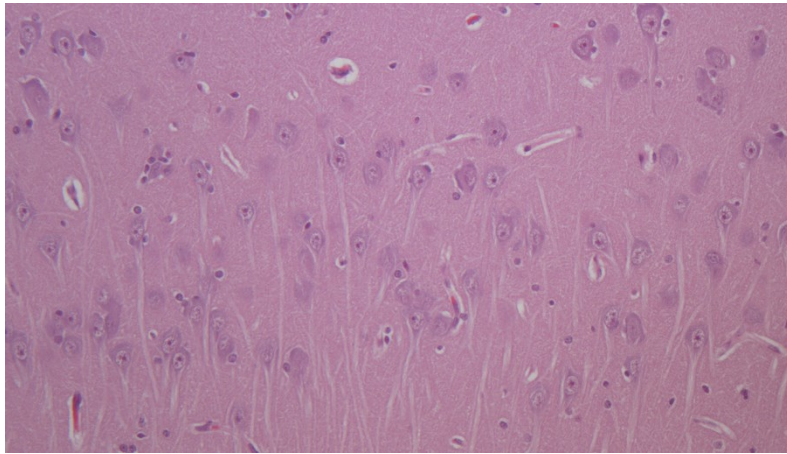

C

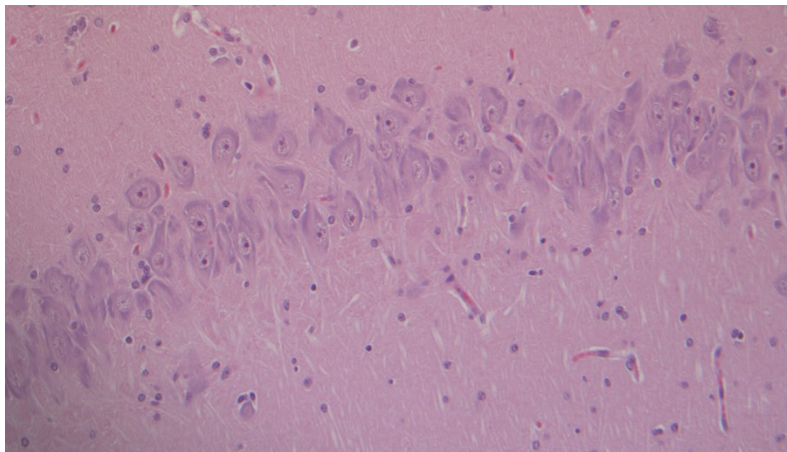

Supplement: Supplemental Information 2 — A, OAI; B, CTR; C, untreated [file peerj-08-10471-s002.pdf]

A

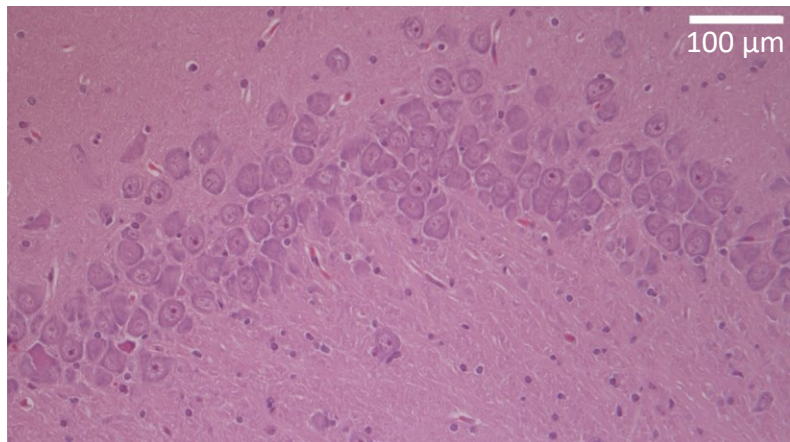

B

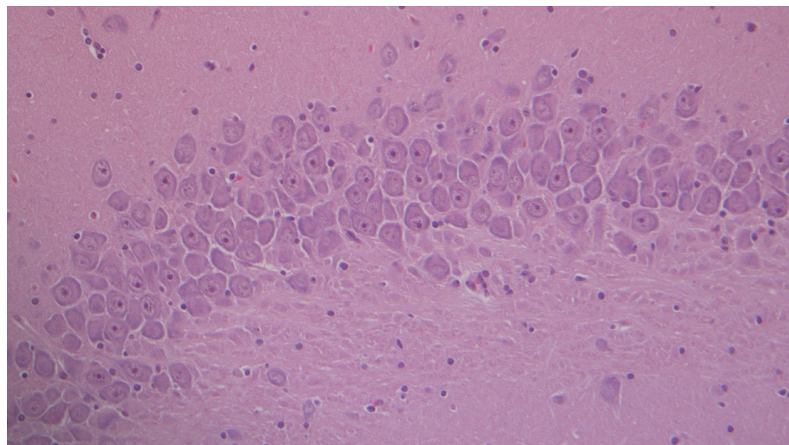

C

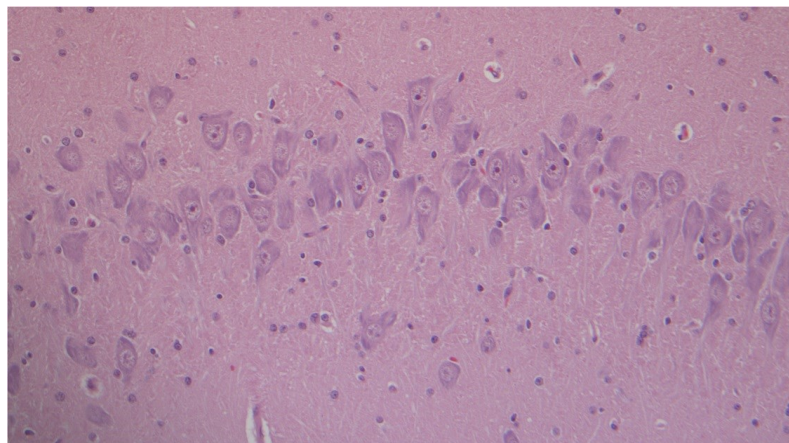

Supplement: Supplemental Information 3 — A, OAI; B, CTR; C, untreated [file peerj-08-10471-s003.pdf]

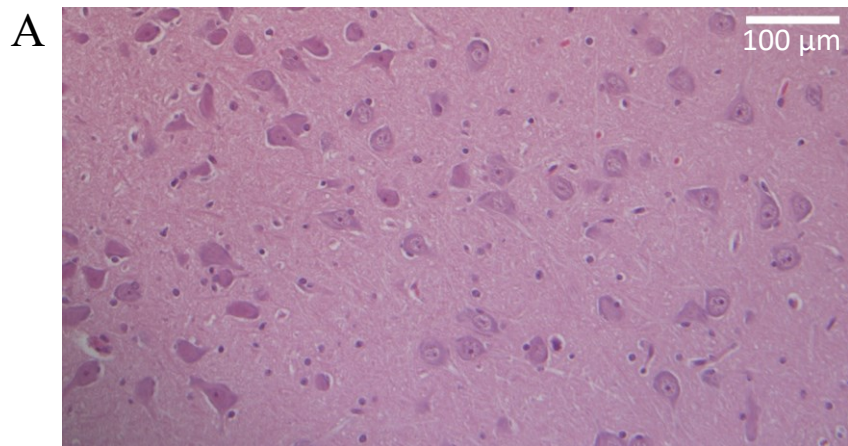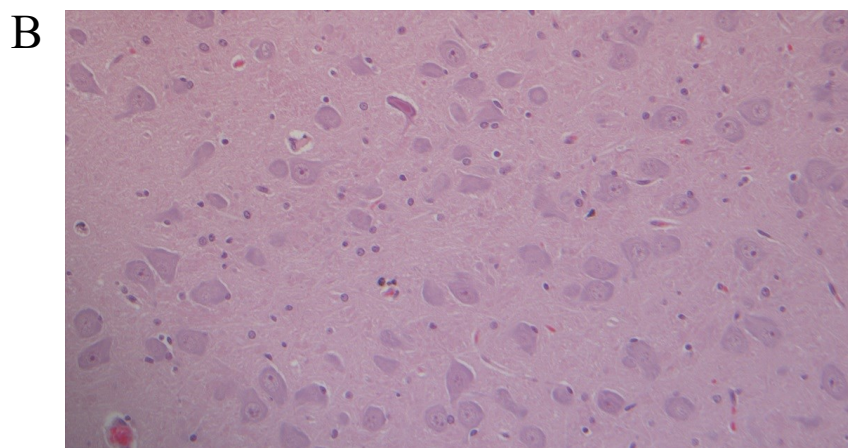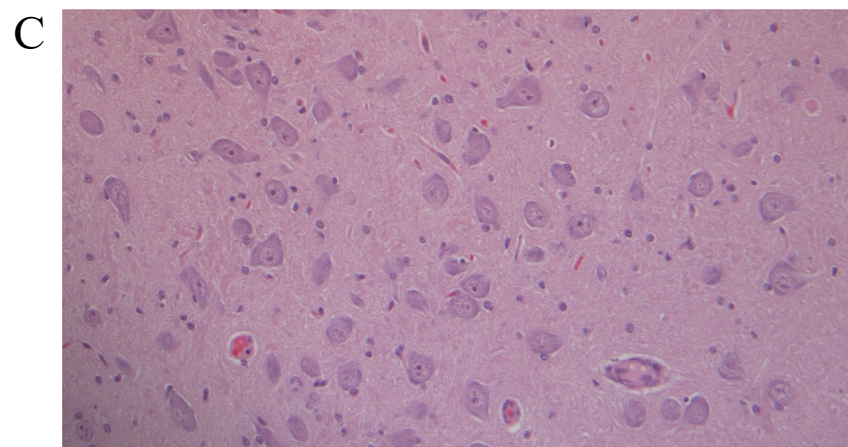

Supplement: Supplemental Information 4 — A, OAI; B, CTR; C, untreated [file peerj-08-10471-s004.pdf]

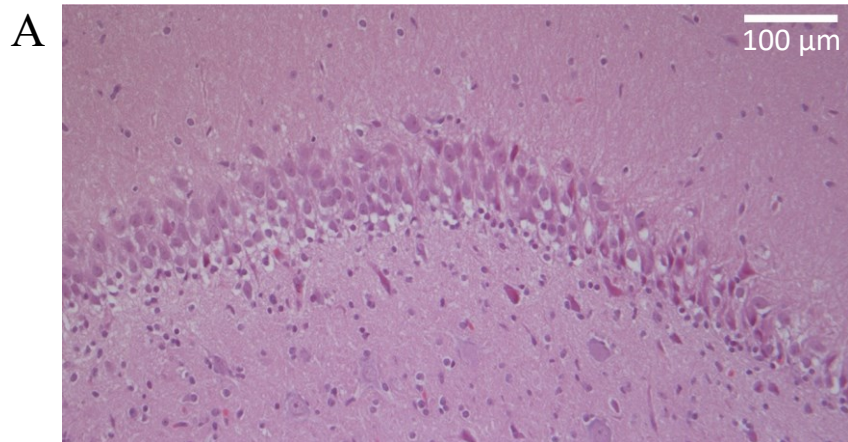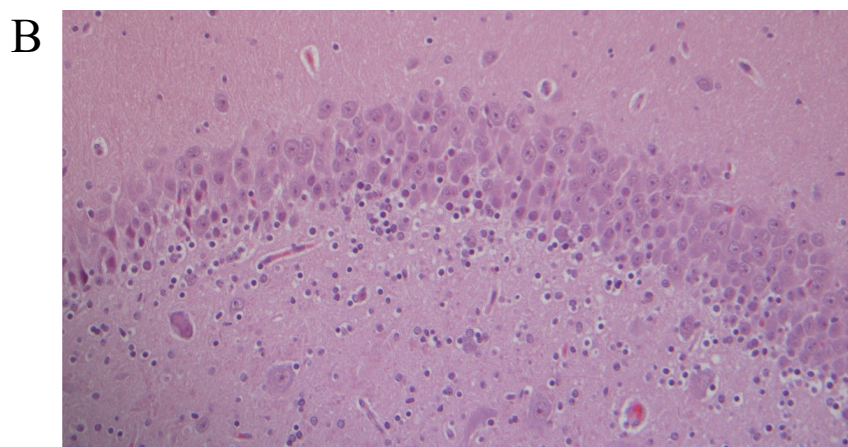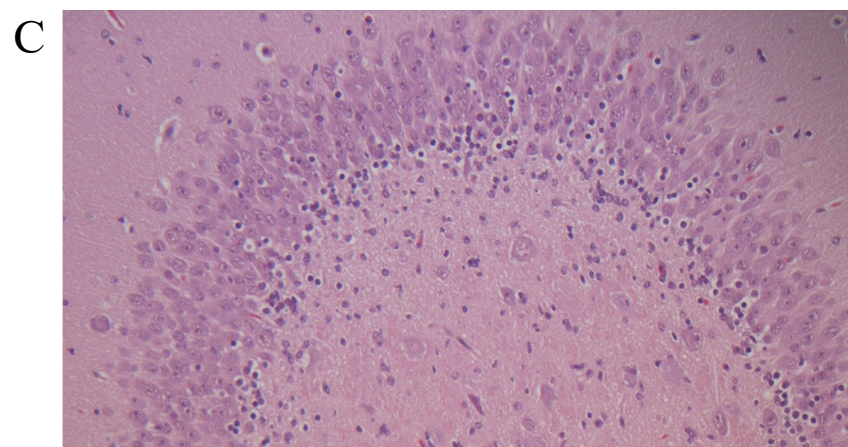

Supplement: Supplemental Information 5 — A, OAI; B, CTR; C, untreated [file peerj-08-10471-s005.pdf]

A

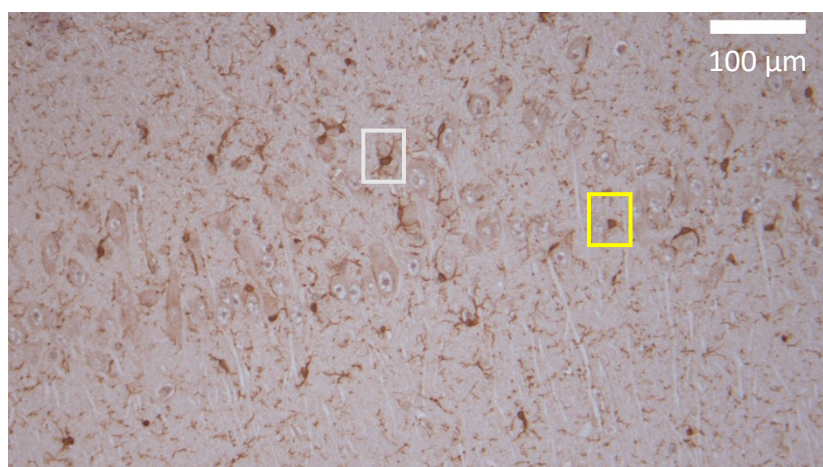

„resting“ microglia cell

„active“ microglia cell

B

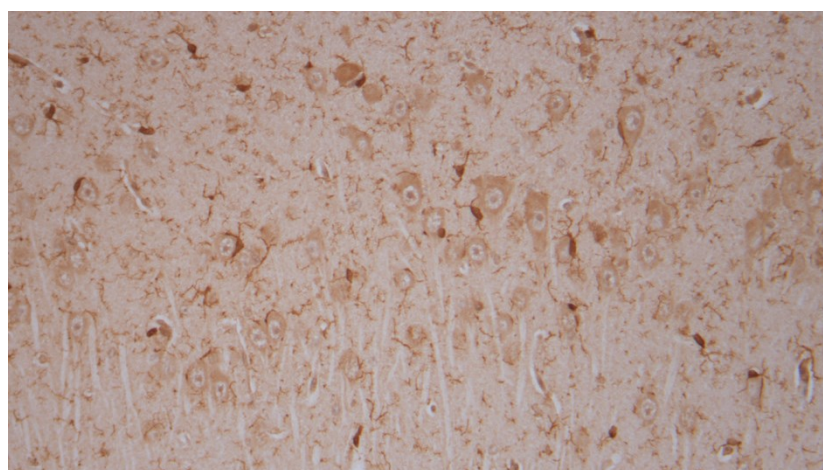

C

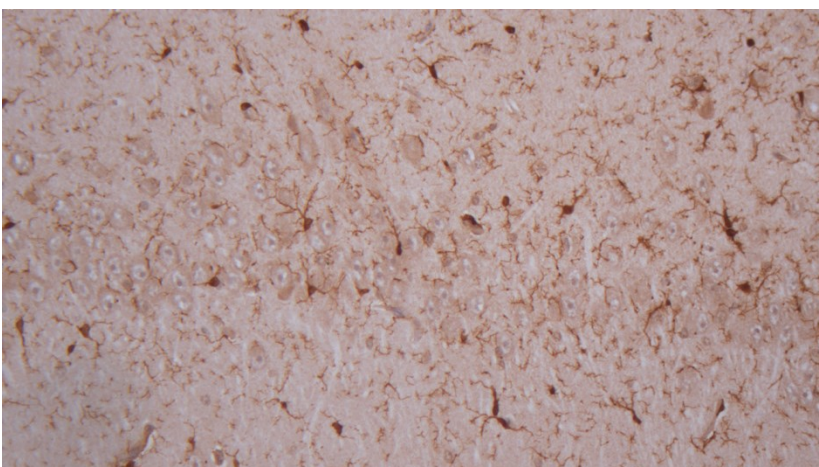

Supplement: Supplemental Information 6 — A, OAI; B, CTR; C, untreated [file peerj-08-10471-s006.pdf]
