# Supplementary material for: Experimental lung injury induces cerebral cytokine mRNA production in pigs
Source: PeerJ. 2020 Dec 9;8:e10471. doi: 10.7717/peerj.10471 (PMC7733330; doi:10.7717/peerj.10471)
Supplement: Supplemental Information 7 [file peerj-08-10471-s007.docx]

|  | **CTR** |  | **OAI** |  |
| --- | --- | --- | --- | --- |
|  | comparison | p-value | comparison | p-value |
| **PEEP** | BLH vs. 18h | 1 | 6h vs. BLH | <0.001 |
| (cm H_2_O) | BLH vs. 12h | 1 | 6h vs. 0h | <0.001 |
|  | BLH vs. 6h | 1 | 6h vs. 18h | 0.004 |
|  | BLH vs. 0h | 1 | 6h vs. 12h | 0.003 |
|  | 0h vs. 18h | 1 | 12h vs. BLH | <0.001 |
|  | 0h vs. 12h | 1 | 12h vs. 0h | 0.074 |
|  | 0h vs. 6h | 1 | 12h vs. 18h | 0.751 |
|  | 6h vs. 18h | 1 | 18h vs. BLH | <0.001 |
|  | 6h vs. 12h | 1 | 18h vs. 0h | 0.06 |
|  | 12h vs. 18h | 1 | 0h vs. BLH | 0.004 |
| **P_peak_** | 18h vs. 0h | 0.106 | 18h vs. BLH | <0.001 |
| (cm H_2_O) | 18h vs. BLH | 0.309 | 18h vs. 12h | 0.159 |
|  | 18h vs. 6h | 0.245 | 18h vs. 0h | 0.197 |
|  | 18h vs. 12h | 0.62 | 18h vs. 6h | 0.217 |
|  | 12h vs. 0h | 0.201 | 6h vs. BLH | <0.001 |
|  | 12h vs. BLH | 0.43 | 6h vs. 12h | 0.659 |
|  | 12h vs. 6h | 0.266 | 6h vs. 0h | 0.62 |
|  | 6h vs. 0h | 0.659 | 0h vs. BLH | <0.001 |
|  | 6h vs. BLH | 0.901 | 0h vs. 12h | 0.71 |
|  | BLH vs. 0h | 0.457 | 12h vs. BLH | <0.001 |
| **V_T_** |  | n.s. |  | n.s. |
| (ml/kg) |  |  |  |  |
| **etCO_2_** |  | n.s. |  | n.s. |
| (mmHg) |  |  |  |  |
| **FiO_2_** | 0h vs. 12h | <0.001 | 0h vs. BLH | <0.001 |
| (%) | 0h vs. 18h | <0.001 | 0h vs. 18h | <0.001 |
|  | 0h vs. 6h | <0.001 | 0h vs. 12h | <0.001 |
|  | 0h vs. BLH | <0.001 | 0h vs. 6h | <0.001 |
|  | BLH vs. 12h | 0.945 | 6h vs. BLH | <0.001 |
|  | BLH vs. 18h | 1 | 6h vs. 18h | <0.001 |
|  | BLH vs. 6h | 1 | 6h vs. 12h | 0.004 |
|  | 6h vs. 12h | 0.845 | 12h vs. BLH | 0.02 |
|  | 6h vs. 18h | 1 | 12h vs. 18h | 0.344 |
|  | 18h vs. 12h | 0.582 | 18h vs. BLH | 0.066 |
| **EVLWI** | 18h vs. BLH | 0.044 | 6h vs. BLH | <0.001 |
| (ml/kg) | 18h vs. 0h | 0.333 | 6h vs. 18h | 0.017 |
|  | 18h vs. 6h | 0.214 | 6h vs. 12h | 0.021 |
|  | 18h vs. 12h | 0.527 | 6h vs. 0h | 0.293 |
|  | 12h vs. BLH | 0.128 | 0h vs. BLH | <0.001 |
|  | 12h vs. 0h | 0.541 | 0h vs. 18h | 0.117 |
|  | 12h vs. 6h | 0.293 | 0h vs. 12h | 0.095 |
|  | 6h vs. BLH | 0.477 | 12h vs. BLH | <0.001 |
|  | 6h vs. 0h | 1 | 12h vs. 18h | 0.751 |
|  | 0h vs. BLH | 0.248 | 18h vs. BLH | <0.001 |
| **Oxygenation** | 0h vs. 18h | <0.001 | BLH vs. 0h | <0.001 |
| **Ratio** | 0h vs. 6h | 0.003 | BLH vs. 6h | <0.001 |
| (mmHg) | 0h vs. 12h | 0.002 | BLH vs. 18h | <0.001 |
|  | 0h vs. BLH | 0.118 | BLH vs. 12h | <0.001 |
|  | BLH vs. 18h | <0.001 | 12h vs. 0h | <0.001 |
|  | BLH vs. 6h | 0.113 | 12h vs. 6h | 0.408 |
|  | BLH vs. 12h | 0.05 | 12h vs. 18h | 0.857 |
|  | 12h vs. 18h | 0.104 | 18h vs. 0h | <0.001 |
|  | 12h vs. 6h | 0.971 | 18h vs. 6h | 0.273 |
|  | 6h vs. 18h | 0.046 | 6h vs. 0h | 0.001 |
