# Supplementary material for: Experimental lung injury induces cerebral cytokine mRNA production in pigs
Source: PeerJ. 2020 Dec 9;8:e10471. doi: 10.7717/peerj.10471 (PMC7733330; doi:10.7717/peerj.10471)
Supplement: Supplemental Information 8 [file peerj-08-10471-s008.docx]

|  | **CTR** |  | **OAI** |  |
| --- | --- | --- | --- | --- |
|  | comparison | p-value | comparison | p-value |
| **HR** | BLH vs. 18h | 0.93 | 0h vs. BLH | 0.002 |
| min^-1^) | BLH vs. 0h | 0.972 | 0h vs. 18h | 0.767 |
|  | BLH vs. 12h | 0.982 | 0h vs. 12h | 0.834 |
|  | BLH vs. 6h | 0.89 | 0h vs. 6h | 0.687 |
|  | 6h vs. 18h | 0.912 | 6h vs. BLH | 0.004 |
|  | 6h vs. 0h | 0.952 | 6h vs. 18h | 0.84 |
|  | 6h vs. 12h | 0.966 | 6h vs. 12h | 0.865 |
|  | 12h vs. 18h | 0.811 | 12h vs. BLH | 0.004 |
|  | 12h vs. 0h | 0.799 | 12h vs. 18h | 0.695 |
|  | 0h vs. 18h | 0.719 | 18h vs. BLH | 0.004 |
| **MAP** |  | n.s. |  | n.s. |
| (mmHg) |  |  |  |  |
| **MPAP** | 12h vs. 0h | 0.013 | 0h vs. BLH | <0.001 |
| (mmHg) | 12h vs. BLH | 0.074 | 0h vs. 18h | <0.001 |
|  | 12h vs. 6h | 0.34 | 0h vs. 12h | <0.001 |
|  | 12h vs. 18h | 0.556 | 0h vs. 6h | <0.001 |
|  | 18h vs. 0h | 0.039 | 6h vs. BLH | <0.001 |
|  | 18h vs. BLH | 0.152 | 6h vs. 18h | 0.057 |
|  | 18h vs. 6h | 0.414 | 6h vs. 12h | 0.04 |
|  | 6h vs. 0h | 0.142 | 12h vs. BLH | <0.001 |
|  | 6h vs. BLH | 0.292 | 12h vs. 18h | 0.803 |
|  | BLH vs. 0h | 0.395 | 18h vs. BLH | <0.001 |
| **CVP** |  | n.s. |  | n.s. |
| (mmHg) |  |  |  |  |
| **PCWP** |  | n.s. |  | n.s. |
| (mmHg) |  |  |  |  |
| **CI** | 12h vs. 0h | 0.838 | 18h vs. BLH | <0.001 |
| (l/min/m^2^) | 12h vs. 18h | 0.751 | 18h vs. 0h | 0.027 |
|  | 12h vs. 6h | 0.806 | 18h vs. 6h | 0.015 |
|  | 12h vs. BLH | 0.556 | 18h vs. 12h | 0.248 |
|  | BLH vs. 0h | 0.971 | 12h vs. BLH | 0.008 |
|  | BLH vs. 18h | 0.914 | 12h vs. 0h | 0.203 |
|  | BLH vs. 6h | 0.973 | 12h vs. 6h | 0.091 |
|  | 6h vs. 0h | 0.912 | 6h vs. BLH | 0.252 |
|  | 6h vs. 18h | 0.712 | 6h vs. 0h | 0.994 |
|  | 18h vs. 0h | 0.97 | 0h vs. BLH | 0.116 |
| **Norepinephrine** | BLH vs. 0h | 1 | 18h vs. BLH | <0.001 |
| (µg/kg/min) | BLH vs. 18h | 1 | 18h vs. 0h | 0.147 |
|  | BLH vs. 6h | 0.993 | 18h vs. 6h | 0.114 |
|  | BLH vs. 12h | 0.945 | 18h vs. 12h | 0.092 |
|  | 12h vs. 0h | 1 | 12h vs. BLH | 0.048 |
|  | 12h vs. 18h | 0.999 | 12h vs. 0h | 0.897 |
|  | 12h vs. 6h | 0.963 | 12h vs. 6h | 0.753 |
|  | 6h vs. 0h | 0.998 | 6h vs. BLH | 0.057 |
|  | 6h vs. 18h | 1 | 6h vs. 0h | 0.899 |
|  | 18h vs. 0h | 0.954 | 0h vs. BLH | 0.03 |
